# The Absence of Interferon-β Promotor Stimulator-1 (IPS-1) Predisposes to Bronchiolitis and Asthma-like Pathology in Response to Pneumoviral Infection in Mice

**DOI:** 10.1038/s41598-017-02564-9

**Published:** 2017-05-24

**Authors:** Jennifer Simpson, Jason P. Lynch, Zhixuan Loh, Vivian Zhang, Rhiannon B. Werder, Kirsten Spann, Simon Phipps

**Affiliations:** 1 School of Biomedical Sciences, The University of Queensland, Queensland, 4072 Australia; 2 Institute for Molecular Biosciences, The University of Queensland, Queensland, 4072 Australia; 3 School of Biomedical Sciences, Queensland University of Technology, 4001 Queensland, Australia; 4 Child Health Research Centre, Institute of Health and Biomedical Innovation, Queensland, 4006 Australia; 5 Australian Infectious Diseases Research Centre, The University of Queensland, Queensland, Australia

## Abstract

Respiratory syncytial virus (RSV)-bronchiolitis is a major cause of infant morbidity and mortality and a risk factor for subsequent asthma. We showed previously that toll-like receptor (TLR)7 in plasmacytoid dendritic cells (pDCs) is critical for protection against bronchiolitis and asthma in mice infected with pneumonia virus of mice (PVM), the mouse homolog of RSV. This lack of redundancy was unexpected as interferon-β promotor stimulator-1 (IPS-1) signalling, downstream of RIG-I-like receptor (RLR) and not TLR7 activation, contributes to host defence in hRSV-inoculated adult mice. To further clarify the role of IPS-1 signalling, we inoculated IPS-1^−/−^ and WT mice with PVM in early-life, and again in later-life, to model the association between bronchiolitis and asthma. IPS-1 deficiency predisposed to severe PVM bronchiolitis, characterised by neutrophilic inflammation and necroptotic airway epithelial cell death, high mobility group box 1 (HMGB1) and IL-33 release, and downstream type-2 inflammation. Secondary infection induced an eosinophilic asthma-like pathophysiology in IPS-1^−/−^ but not WT mice. Mechanistically, we identified that IPS-1 is necessary for pDC recruitment, IFN-α production and viral control. Our findings suggest that TLR7 and RLR signalling work collaboratively to optimally control the host response to pneumovirus infection thereby protecting against viral bronchiolitis and subsequent asthma.

## Introduction

Asthma is a chronic inflammatory disease characterised by airway hyperreactivity (AHR), type-2 inflammation, and airway remodelling, which includes mucous cell hyperplasia, airway smooth muscle (ASM) growth, and the deposition of matricellular proteins (e.g. periostin). In addition to aeroallergen exposure, type-2 inflammation in the airways can occur in response to respiratory viruses such as respiratory syncytial virus (RSV), particularly during wheezy episodes in early-life^1, 2^. Indeed, several prospective birth cohort studies have shown that severe RSV-bronchiolitis is a major independent risk factor for subsequent asthma^3–7^. The association between bronchiolitis severity and asthma risk^5, 6^, together with preliminary findings from studies using Palivizumab to inhibit RSV infection^8^, lend weight to the argument that RSV bronchiolitis is causal for asthma. However, this topic remains highly contentious, and definitive proof of causality awaits the arrival of an effective RSV vaccine. Nevertheless, it is likely that genetic defects and/or environmental stimuli that negatively affect the host response to RSV infection in early-life also increase the risk of asthma development later in life^9^.

The host response to infection is triggered by the activation of one or more pattern recognition receptors (PRRs), leading to the production of various cytokines and chemokines that both orchestrate the innate immune response, and shape the nature of the adaptive response. Unsurprisingly, genetic variants of various virus-sensing PRRs and anti-viral mediators are associated with an increased risk of severe RSV bronchiolitis^10–12^. This includes members of the endosomal toll-like receptor family such as *TLR7* ^12^ and members of the type-I IFN family such as *IFNA5* ^13^. Additionally, polymorphisms in retinoic acid inducible gene 1 (*RIGI)*, a member of the RNA-helicase like receptors [RLRs]^14, 15^, and *IPS1* ^16^ (IFN-β promoter stimulator 1, alternatively named MAVS: mitochondrial antiviral-signalling protein/virus-induced signalling adapter), an intracellular adapter protein that mediates RLR signalling, are linked to type-I IFN deficiency in man and may therefore underlie susceptibility to viral infections. Using pneumonia virus of mice (PVM), a mouse pneumovirus that replicates the more severe forms of hRSV in children^17–19^, we have previously shown that neonatal TLR7^−/−^ mice develop severe bronchiolitis. Moreover, PVM challenge of TLR7^−/−^ mice in later-life induces an asthma-like pathology including type-2 cytokine production, airway eosinophilia and ASM remodelling^17^. Intriguingly, the adoptive transfer of TLR7-competent pDCs to TLR7^−/−^ mice in early-life is sufficient to reconstitute the antiviral response, as highlighted by the restoration of type I and III IFN production and accelerated viral clearance, suggesting that TLR7 signalling in pDCs is critical for protection against severe bronchiolitis. This lack of redundancy in PRR signalling was unexpected, and appears to contradict work by other investigators who have shown that IPS-1, essential for RLR but not TLR signalling, is necessary for the production of type I IFN (α/β) in adult mice following inoculation with hRSV^20–22^. Following infection with an RNA virus, IPS-1 signalling is necessary for type-I IFN production in most cell types with the exception of pDCs^23^, and hence one possible explanation for the apparent redundancy of function amongst TLR7 and the RLRs during pneumovirus infection would be that the two systems work collaboratively to optimally control the host response^24^. Alternatively, the different phenotypes might reflect the limitations of infecting mice with a human pathogen, or the age at which the mice were inoculated, which can have profound effects on the nature of the immune response^25^. To assess these possibilities, we explored whether IPS-1 deficiency predisposes toward the development of severe bronchiolitis and subsequent asthma-like pathologies following primary and secondary infection with PVM respectively.

## Results

### Neonatal IPS-1^**−**/**−**^ mice are more susceptible to acute infection with PVM

**Figure 1:**
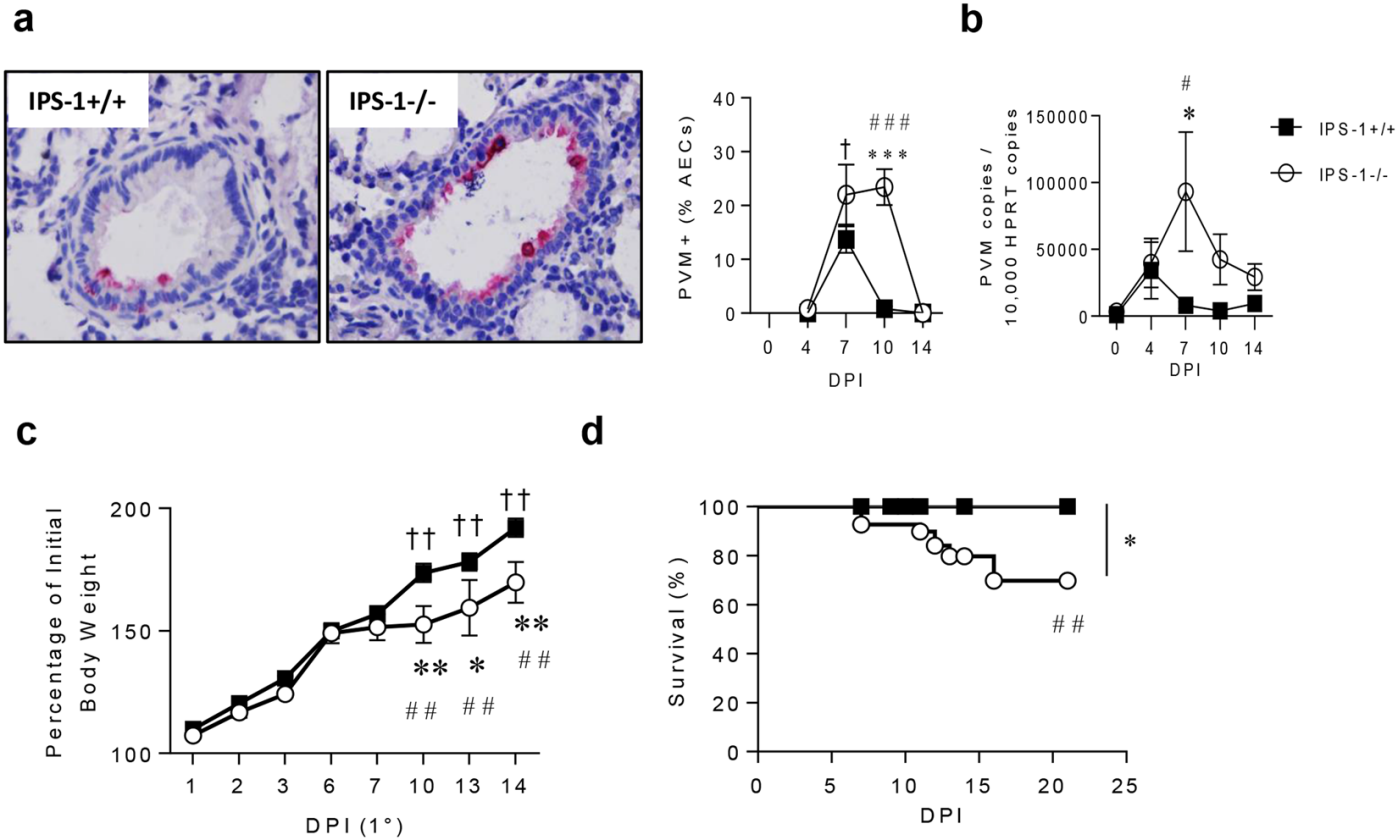
Absence of IPS-1 predisposes towards elevated viral load and increased morbidity and mortality during acute viral infection. (**a**) Representative micrograph (x1000 magnification) of PVM immunoreactivity in the airway epithelium, quantification of PVM positive AECs and (**b**) quantitative PCR of PVM copy number in lung. (**c**) Percentage of total body weight. (**d**) Survival of WT and IPS-1^−/−^ mice represented as percentage. Vehicle control mice were sacrificed on day 7 of life (i.e. 0 dpi). Experiments were performed twice with 5 to 7 mice per group. Data are mean and the standard error of the mean. *^,^**^,^***^,^****Denotes significance between WT and IPS-1^−/−^ infected mice. ^#,##,###^Denotes significance between infected and vehicle treated IPS-1^−/−^ mice. ^†,††^Denotes significance between vehicle and infected WT mice.

Severe bronchiolitis is a major cause of infant morbidity and mortality^1^. To determine whether the absence of IPS-1 would increase the severity of viral disease, WT and IPS-1^−/−^ neonatal mice were intranasally inoculated with a low inoculum (2 pfu) of PVM or vehicle (DMEM/10% FCS) at 7 days of age and killed at 4, 7, 10 and 14 days post primary infection (dpi). Viral load in the airway epithelium, the primary site of PVM infection^17–19^, peaked at 7 dpi and decreased to undetectable levels by 10 dpi in WT mice (Fig. 1a). In contrast, the absence of IPS-1 led to a significant increase in viral load and delayed clearance from the airway epithelium until 14 dpi (Fig. 1a). Similarly, PVM *SH* gene copy number, indicative of viral replication, was significantly increased in IPS-1^−/−^ compared to WT mice at 7 dpi (Fig. 1b). Loss of viral control in IPS-1^−/−^ mice was associated with stunted weight gain from 7–14 dpi (Fig. 1c), and strikingly, approximately one third of the IPS-1^−/−^ cohort died in this period (Fig. 1d).

### The absence of IPS-1 increases neutrophilic inflammation and airway epithelial cell death

**Figure 2:**
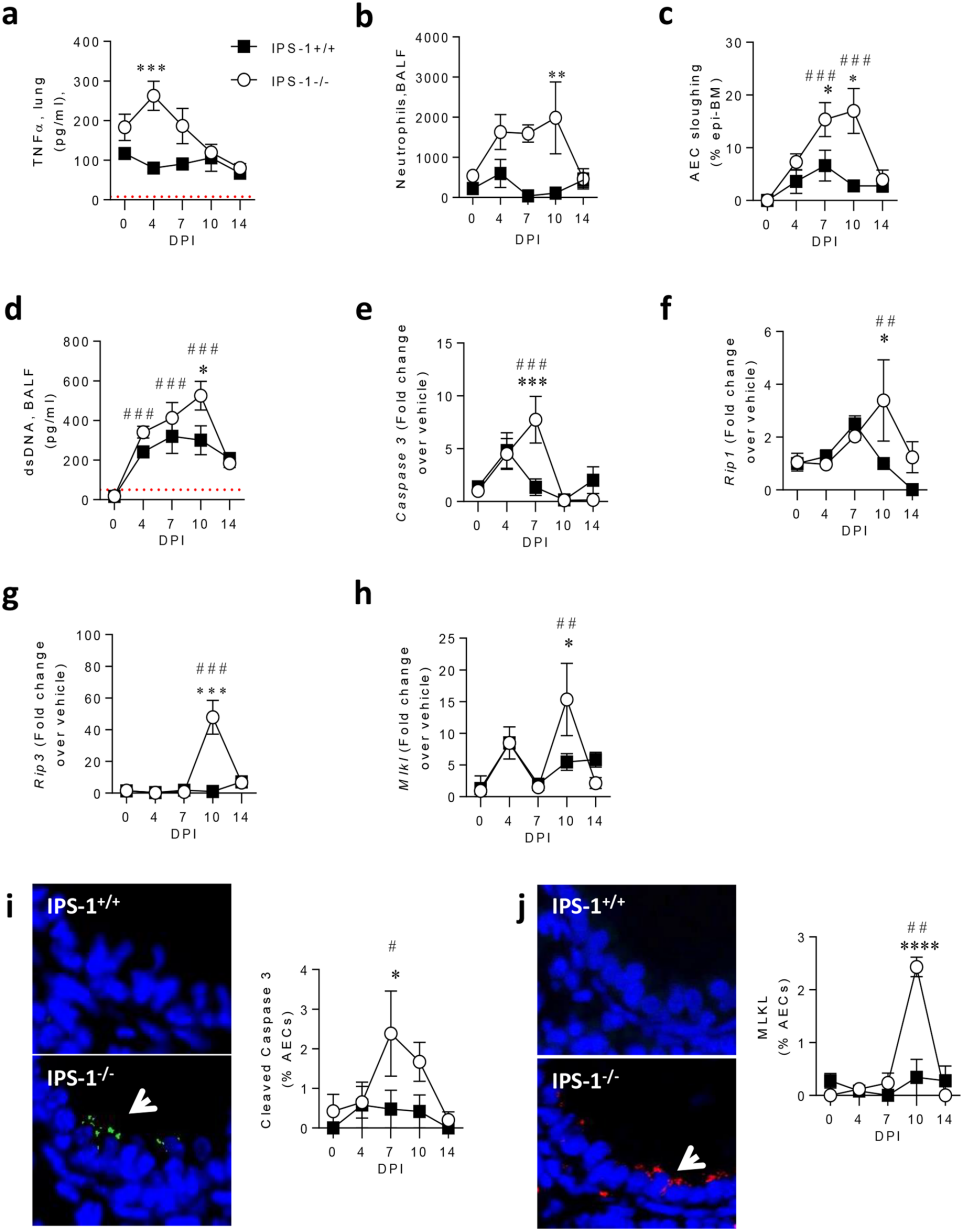
Absence of IPS-1 predisposes towards bronchiolitis and airway epithelial cell necroptosis during acute viral infection. (**a**) TNF-α protein expression in lung. (**b**) Neutrophil numbers in bronchoalveolar lavage fluid (BALF). (**c**) AEC sloughing represented as percentage of airway basement membrane. (**d**) dsDNA expression in BALF. Lung gene expression of (**e**) *Caspase 3*, (**f**) *Rip1*, (**g**) *Rip3 and* (**h**) *MLKL*. (**i**) Left panel: representative micrograph (100x magnification) of cleaved caspase 3 immunostaining. Right panel: Quantification of cleaved caspase 3 expression in AECs. (**j**) Left panel: representative micrograph (100x magnification) of MLKL immunostaining. Right panel: Quantification of cleaved MLKL expression in AECs. Experiments were performed twice with 6 to 8 mice per group. Vehicle control mice were sacrificed on day 7 of life (i.e. 0 dpi). Data are mean and the standard error of the mean. *^,^**^,^***^,^****Denotes significance between WT and IPS-1^−/−^ infected mice. ^#,##,###^Denotes significance between infected and vehicle IPS-1^−/−^ mice. Detection limits are denoted by a red dotted line.

Stunted weight gain typically stems from a hyper-inflammatory response. PVM infection significantly increased the expression of TNF-α, a pro-inflammatory mediator and inducer of cell death, at 4 dpi in IPS-1^−/−^ compared to WT mice (Fig. 2a). Neutrophilic inflammation in the airways was slightly elevated in both mouse strains at 4 dpi, but was significantly greater in IPS-1^−/−^ mice compared to WT mice at 10 dpi (Fig. 2b). AEC cell death and sloughing forms part of the host response to limit viral replication and spread, and as expected, AEC sloughing was elevated in WT mice, peaking at 7 dpi. However, in IPS-1^−/−^ mice, the degree of AEC sloughing was far more pronounced, most notably at 7 and 10 dpi when it was significantly greater than in WT mice (Fig. 2c). AEC sloughing is a hallmark feature of severe bronchiolitis^26, 27^, and depending on the mode of cell death, can lead to the release of various damage-associated molecular patterns. Notably the levels of dsDNA in the airway mirrored the degree of AEC sloughing in both strains, and were significantly greater in IPS-1^−/−^ mice (Fig. 2d). We next questioned whether cell death was mediated by apoptosis (i.e. caspase-3-dependent) and/or via the activation of necroptosis, typically associated with the kinases *Rip1* (receptor-interacting serine/threonine-protein kinase 1), *Rip3* and *Mlkl* (mixed lineage kinase domain-like protein). In both WT and IPS-1^−/−^ mice there was a rapid increase in lung *Caspase-3* expression at 4 dpi, however, whereas this response declined at 7 dpi in WT mice, it was further increased in IPS-1^−/−^ mice, before waning at 10 dpi (Fig. 2e). PVM infection had little effect on the expression of *Rip1*, *Rip3* and *Mlkl* in the lungs of WT mice, however, all three genes were significantly elevated in IPS-1^−/−^ mice at 10 dpi (Fig. 2f–h). To identify which cells were dying we probed the lungs for cleaved (activated) caspase-3 and MLKL by immunohistochemistry. In IPS-1^−/−^ mice, cleaved caspase-3 and MLKL were both exclusively expressed in the airway epithelium although cleaved caspase-3 was significantly increased at 7 dpi, while MLKL was significantly increased at 10 dpi (Fig. 2i,j), consistent with the pattern of mRNA expression of both genes. Collectively, these data show that the absence of IPS-1 increases at least two modes of cells death and that these occur in a temporally distinct manner.

### Severe bronchiolitis in IPS-1^**−**/**−**^ mice is associated with alarmin release and type-2 inflammation

**Figure 3:**
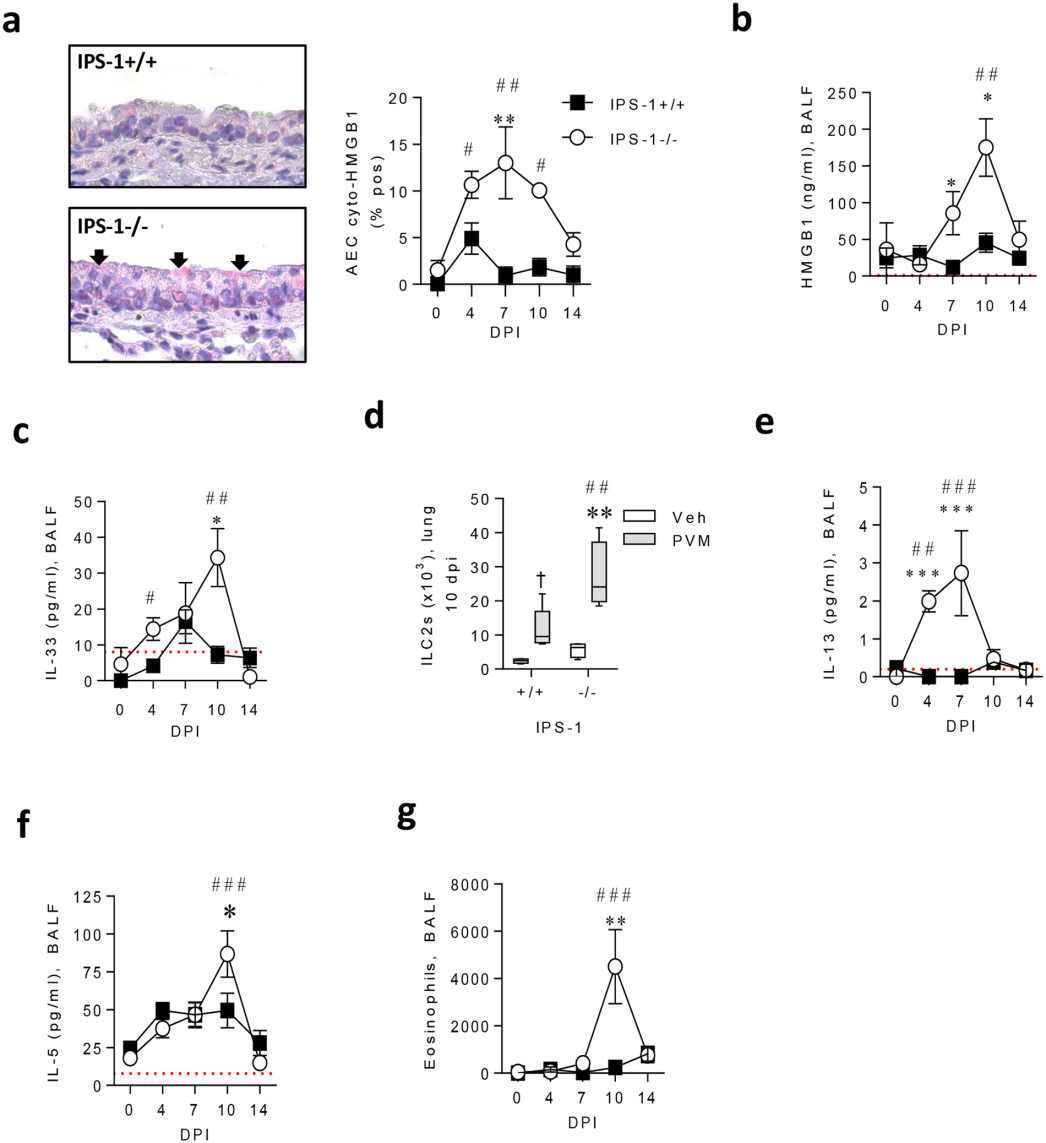
Absence of IPS-1 predisposes towards alarmin release and type-2 inflammation during acute viral infection. (**a**) Left panel: representative micrograph (40x magnification) of HMGB1 immunostaining. Right panel: Cytoplasmic AEC expression of HMGB1. (**b**) HMGB1 protein expression in BALF. (**c**) IL-33 protein expression in BALF. (**d**) Number of ILC2s in lung at 10 dpi (gated on Lin^−^, CD25^+^, ST2^+^, CD90.2^+^). (**e**) IL-13 protein expression in BALF. (**f**) IL-5 protein expression in BALF. (**g**) Eosinophil number in BALF. Vehicle treated control mice were sacrificed on day 7 of life (i.e. 0 dpi). Experiments were performed twice with 5 to 7 mice per group. Data are mean and the standard error of the mean. *^,^**^,^***^,^****Denotes significance between WT and IPS-1^−/−^ infected mice. ^#,##,###^Denotes significance between infected and vehicle treated IPS-1^−/−^ mice. Detection limits are denoted by a red dotted line.

Airway epithelial cell injury or death can induce the nuclear-to-cytoplasmic translocation and extracellular release of the nuclear alarmins HGMB1 and IL-33, both implicated in the induction of type-2 inflammation^19, 28, 29^. In WT mice, levels of cytoplasmic HMGB1 were slightly elevated at 4 dpi, whereas in IPS-1^−/−^ mice, the proportion of AECs expressing cytoplasmic HMGB1 was significantly elevated between 4 and 10 dpi, and significantly greater than WT mice at 7 dpi (Fig. 3a). This phenotype preceded the increase in extracellular HGMB1 levels detected in the airway lumen at 7 and 10 dpi in IPS-1^−/−^ mice (Fig. 3b). Similarly, IL-33 levels were significantly elevated in IPS-1^−/−^ but not WT mice, occurring as early as 4 dpi, with levels significantly different between the two strains at 10 dpi (Fig. 3c). At this time point, the number of type-2 innate lymphoid cells (ILC2s) in the lungs was elevated in both strains, and significantly greater in IPS-1^−/−^ compared to WT mice (Fig. 3d and Supplement 1). Other cytokines known to activate ILC2s, such as thymic stromal lymphopoietin and IL-25 were not elevated in response to PVM infection in either mouse strain (data not shown). The expression of the type-2 cytokine IL-13 was significantly elevated at 4 and 7 dpi in IPS-1^−/−^ but not WT mice (Fig. 3e), whereas IL-5 was significantly greater at 10 dpi (Fig. 3f). Consistent with the production of type-2 cytokines, there was a pronounced airway eosinophilia at 10 dpi in IPS-1^−/−^ but not WT mice (Fig. 3g). In contrast, the numbers of monocytes and lymphocytes in the BAL did not differ between WT and IPS-1^−/−^ mice (data not shown). In an attempt to address whether the type-2 inflammatory response of IPS-1^−/−^ neonates was dependent on the age at first infection, we inoculated adult mice with 2 PFU and analysed disease parameters at 7 dpi. In contrast to the primary infection in neonatal mice, we did not observe an increase in eosinophil numbers (Supplement 2a) or HMGB1 expression in these mice (Supplement 2b), suggesting that the predisposition of IPS-1^−/−^ mice to virus-induced type-2 inflammation is age-dependent.

### Type-2 inflammation is accompanied by airway remodelling in bronchiolitic IPS-1^**−**/**−**^ mice

**Figure 4:**
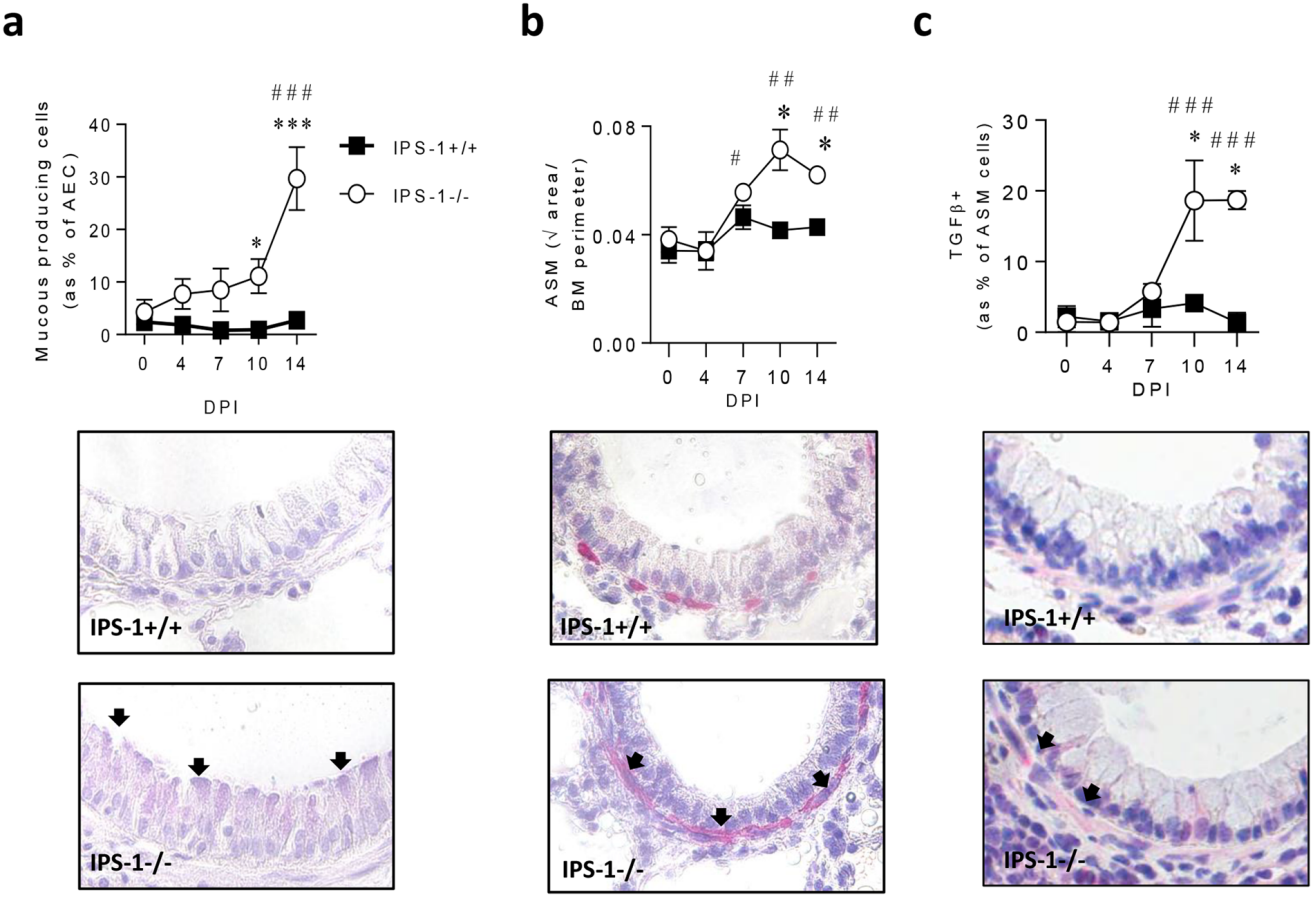
IPS-1^−/−^ mice exhibit elevated airway remodelling in acute viral infection. (**a**) Top panel: Number of mucous-producing AECs. Bottom panel: representative micrograph (40x magnification) of Periodic acid–Schiff staining. (**b**) Top panel: Airway smooth muscle (ASM) area. Bottom panel: representative micrograph (40x magnification) of alpha actin smooth muscle immunostaining. (**c**) Top panel: Percentage of ASM cells expressing TGFβ. Bottom panel: representative micrograph (40x magnification) of TGFβ immunostaining. Vehicle control mice were sacrificed on day 7 of life (i.e. 0 dpi). Experiments were performed twice with 5 to 7 mice per group. Data are mean and the standard error of the mean. *^,^**^,^***^,^****Denotes significance between WT and IPS-1^−/−^ infected mice. ^#,##,###^Denotes significance between infected and vehicle IPS-1^−/−^ mice.

Type-2 cytokines, particularly IL-13, can elicit muocus hypersecretion and ASM remodelling^30, 31^, which is now recognised to occur in preschool aged children who are later diagnosed with asthma^32^. In WT mice, PVM infection did not increase the proportion of mucous producing AECs, whereas mucous cell hyperplasia was significantly increased in IPS-1^−/−^ mice at 10 and 14 dpi (Fig. 4a). This was accompanied by an increase in ASM area surrounding the small airways at 10 and 14 dpi (Fig. 4b), and a higher proportion of ASM cells expressing the smooth muscle mitogen TGFβ in IPS-1^−/−^ but not WT mice (Fig. 4c).

### Secondary infection of IPS-1^**−**/**−**^ mice increases type-2 inflammation

**Figure 5:**
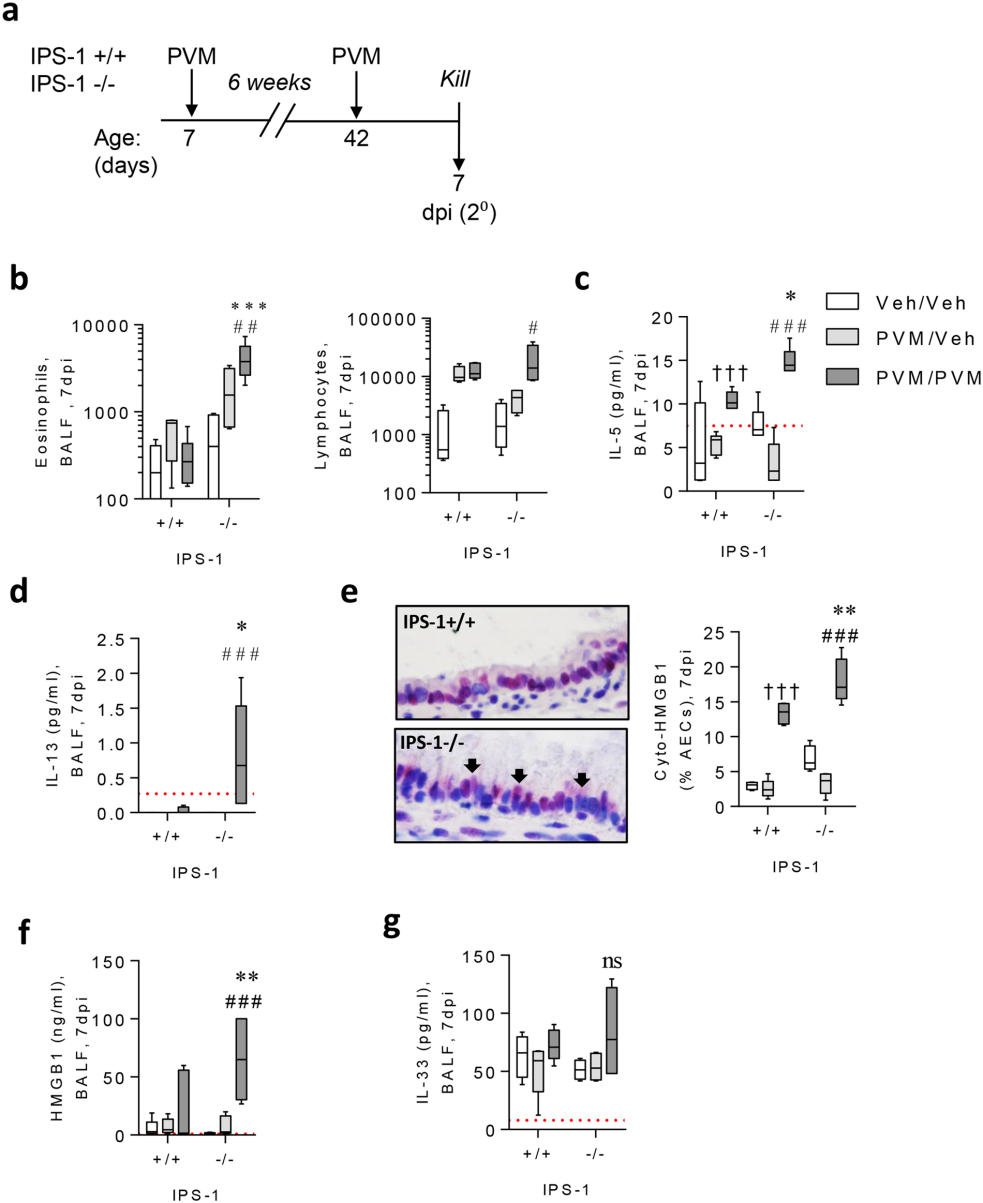
Viral challenge of IPS-1^−/−^ mice induces a mild Th2 phenotype. (**a**) Study design, 1 week old neonatal WT or IPS-1^−/−^ mice were infected with 2 pfu in early life or vehicle diluent and were reinfected with 100 pfu or vehicle diluent 6 weeks post primary infection. Mice were euthanised at 7 days post reinfection. (**b**) Eosinophil and lymphocyte numbers in BALF. (**c**) IL-5 protein expression in BALF. (**d**) IL-13 protein expression in BALF. (**e**) Left panel: representative micrograph (40x magnification) of HMGB1 immunostaining. Right panel: Cytoplasmic AEC expression of HMGB1. (**f**) HMGB1 protein expression in BALF. (**g**) IL-33 protein expression in BALF. Vehicle control mice are denoted as Veh/Veh. Experiments were performed twice with 5 to 7 mice per group. Data are mean and the standard error of the mean. *^,^**^,^***^,^****Denotes significance between WT and IPS-1^−/−^ infected mice. ^#,##,###^Denotes significance between infected and vehicle IPS-1^−/−^ mice. ^†,††,†††^Denotes significance between vehicle and infected WT mice. Detection limits are denoted by a red dotted line.

Severe bronchiolitis is a major independent risk factor for subsequent asthma^5, 6^. Hence, we hypothesised that IPS-1^−/−^ mice that had developed bronchiolitis in early-life would be predisposed toward virus-induced asthma-like pathologies upon re-exposure to PVM in later-life. To test this, 7 week old mice that had developed severe viral bronchiolitis (IPS-1^−/−^) or had been asymptomatic (WT neonates) in early-life were re-challenged with PVM and euthanized at 3 or 7 dpi (Fig. 5a). Non-infected mice served as controls. In response to viral challenge, IPS-1^−/−^ mice developed a type-2 inflammatory response as indicated by a significant eosinophilia and lymphoplasia (Fig. 5b), together with increased production of IL-5 (Fig. 5c) and IL-13 (Fig. 5d) in bronchoalveolar lavage fluid (BALF). In contrast, type-1 (IFN-γ) and type-17 (IL-17A) associated cytokines, as well as neutrophil numbers, were unchanged in IPS-1^−/−^ mice (Supplement 3). Similar to the primary infection, the proportion of AECs with cytoplasmic HMGB1 and levels of HMGB1 in BALF were significantly elevated in infected compared to vehicle-inoculated IPS-1^−/−^ mice, and were significantly greater than in WT mice (Fig. 5e,f). Additionally, IL-33 levels were elevated in infected compared to vehicle-inoculated IPS-1^−/−^ mice, although the difference between infected WT and IPS-1^−/−^ mice was not significant (Fig. 5g).

### Secondary infection of IPS-1^−/−^ mice increases AHR and features of airway remodelling associated with a non-atopic asthma-like phenotype

**Figure 6:**
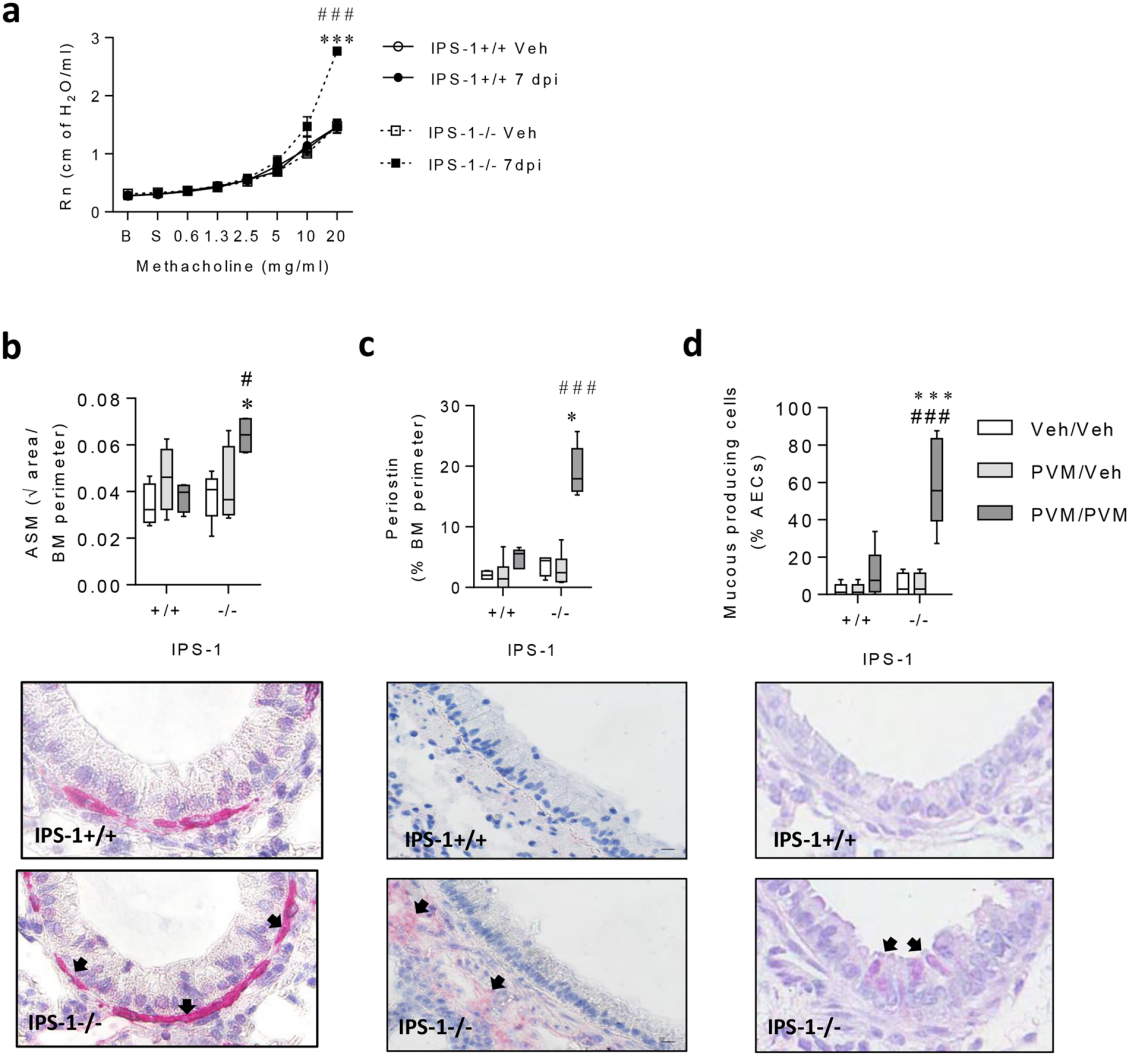
Viral challenge of IPS-1^−/−^mice predisposes to AHR and airway remodelling. (**a**) Central airways resistance was measured in response to increasing doses of nebulized methacholine. B, Baseline. S, Saline. (**b**) Top panel: Airway smooth muscle (ASM) area. Bottom panel: representative micrograph (40x magnification) of alpha actin smooth muscle immunostaining. (**c**) Top panel: Airway perimeter periostin expression. Bottom panel: representative micrograph (40x magnification) of periostin immunostaining. (**d**) Top panel: Number of mucous-producing AECs. Bottom panel: representative micrograph (40x magnification) of Periodic acid–Schiff staining. Vehicle control mice are denoted as Veh/Veh. Experiments were performed twice with 5 to 9 mice per group. Data are mean and the standard error of the mean. *^,^**^,^***^,^****Denotes significance between WT and IPS-1^−/−^ infected mice. ^#,##,###^Denotes significance between infected and vehicle IPS-1^−/−^ mice. ^†,††,†††^Denotes significance between vehicle and infected WT mice.

Since type-2 inflammation was increased in IPS-1^−/−^ mice following viral challenge, we next determined whether these mice developed asthma-like pathologies, including AHR and airway remodelling. In WT mice, airway resistance in response to escalating doses of methacholine was the same between the vehicle and PVM infected groups. In contrast, airway resistance was significantly greater in PVM challenged compared to vehicle-challenged IPS-1^−/−^ mice or PVM challenged WT mice (Fig. 6a). Similarly, ASM mass (Fig. 6b), periostin deposition (Fig. 6c), and mucus cell metaplasia (Fig. 6d) were not increased in WT mice following PVM challenge, but all of these pathological features were significantly elevated in IPS-1^−/−^ mice in response to PVM challenge. Notably, these pathologies were not present in IPS-1^−/−^ mice that were infected as neonates and killed at the time of viral challenge in adulthood (Fig. 6b–d), and hence, were not simply a consequence of the primary infection alone.

### IPS-1 regulates pDC-mediated antiviral immunity

**Figure 7:**
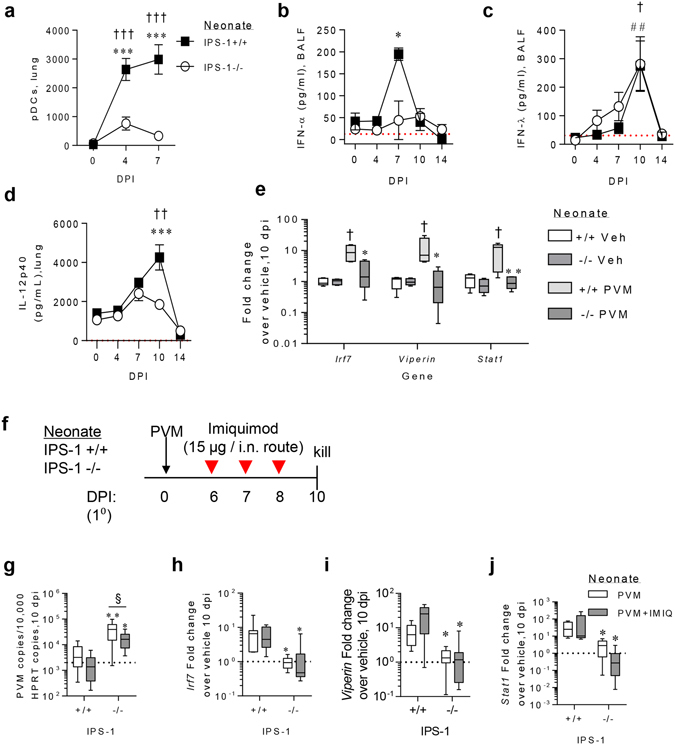
IPS-1 is critical for pDC recruitment and IFN-α production in response to acute viral infection in early life. (**a**) pDC number in lung at 4 and 7 dpi (gated on CD11b^-^ B220^+^ CD11c^+^ Siglec-H^+^ CD45RA^+^). (**b**) IFN-α protein expression in BALF. (**c**) IFN-λ protein expression in BALF. (**d**) IL-12p40 protein expression in lung. (**e**) IFN-stimulated gene expression in lung at 10 dpi. (**j**) Study design for imiquimod (IMIQ) treatment of WT and IPS-1^−/−^ mice. (**g**) Quantitative PCR of PVM copy number in lung. Lung gene expression of *Irf7* (**h**), *Viperin* (**i**) *and Stat1* (**j**). Experiments were performed twice with 8 to 12 mice per group. Vehicle control mice were sacrificed on day 7 of life (i.e. 0 dpi). Data are mean and the standard error of the mean. *^,^**^,^***^,^****Denotes significance compared to WT infected mice. ^§^Denotes significance between imiquimod treated and vehicle (PBS) treated mice. Detection limits are denoted by a red dotted line. The average mRNA expression for WT vehicle mice are denoted by a black dotted line.

Because the IPS-1^−/−^ mice phenocopied the response observed previously in TLR7^−/−^ mice to primary and secondary PVM infection^17, 18^, we next questioned whether IPS-1 deficiency affected the recruitment of pDCs in the primary infection. As expected, PVM infection of WT mice led to a significant increase in the number of pDCs in the lungs at both 4 and 7 dpi (Fig. 7a). However, in the absence of IPS-1, the numbers of pDCs following PVM infection were not significantly different to the vehicle control group. The failure of IPS-1^−/−^ mice to increase pDCs in the lung was associated with an attenuated antiviral cytokine response, most notably in the production of IFN-α at 7 dpi. In response to PVM infection, IFN-α levels in the BALF increased 5-fold in WT mice whereas there was no significant increase in IPS-1^−/−^ mice (Fig. 7b). By contrast, the production of other IFNs, namely IFN-λ and IFN-γ, was similarly elevated following PVM infection in both WT and IPS-1^−/−^ mice (Fig. 7c and data not shown). Like IFN-α, the expression of IL-12p40 was significantly increased in WT mice but not IPS-1^−/−^ mice following PVM infection, and at 10 dpi, IL-12p40 levels were significantly lower in PVM infected IPS-1^−/−^ mice compared to WT mice (Fig. 7d). The elevated IFN-α production was associated with a significant increase in the expression of IFN-stimulated genes, including interferon regulatory factor (*Irf)7*, *Viperin* and signal transducer and activator of transcription (*Stat*)1, in the lungs of WT mice at 10 dpi (Fig. 7e). However, these genes were not up-regulated in the absence of IPS-1, and their expression was significantly lower in IPS-1^−/−^ mice compared to WT mice infected with PVM. Taken together, these data suggest that IPS-1 mediated signalling contributes to the recruitment of pDCs, and as a consequence, plays an important role in the generation of the antiviral state.

In an attempt to address whether the defect in pDC recruitment affected TLR7 responsiveness in IPS-1-deficient mice, we inoculated WT and IPS-1 KO mice with PVM then exposed the mice to imiquimod, a TLR7 agonist, at 6, 7 and 8 dpi (around the spike in IFN-α production in WT mice; Fig. 7b) and euthanized the mice at 10 dpi (Fig. 7f). TLR7 stimulation significantly decreased viral load in IPS-1^−/−^ mice, although the magnitude of this response was small. Consequently, the viral load in imiquimod-treated IPS-1^−/−^ mice remained significantly higher than WT neonates (Fig. 7g). Interestingly, TLR7 stimulation of PVM infected IPS-1^−/−^ mice failed to increase n *Irf7* (Fig. 7h), *Viperin* (Fig. 7i) or *Stat1* (Fig. 7j) mRNA expression, and thus their levels remained significantly lower than that of PVM infected WT neonates. Although not definitive, these data support the idea that TLR7 and IPS-1 signalling work in a linear manner to induce the antiviral response to PVM.

### TLR7/IPS-1-induced IFN-α production negatively regulates type-2 inflammation in PVM infected IPS-1^−/−^ mice

**Figure 8:**
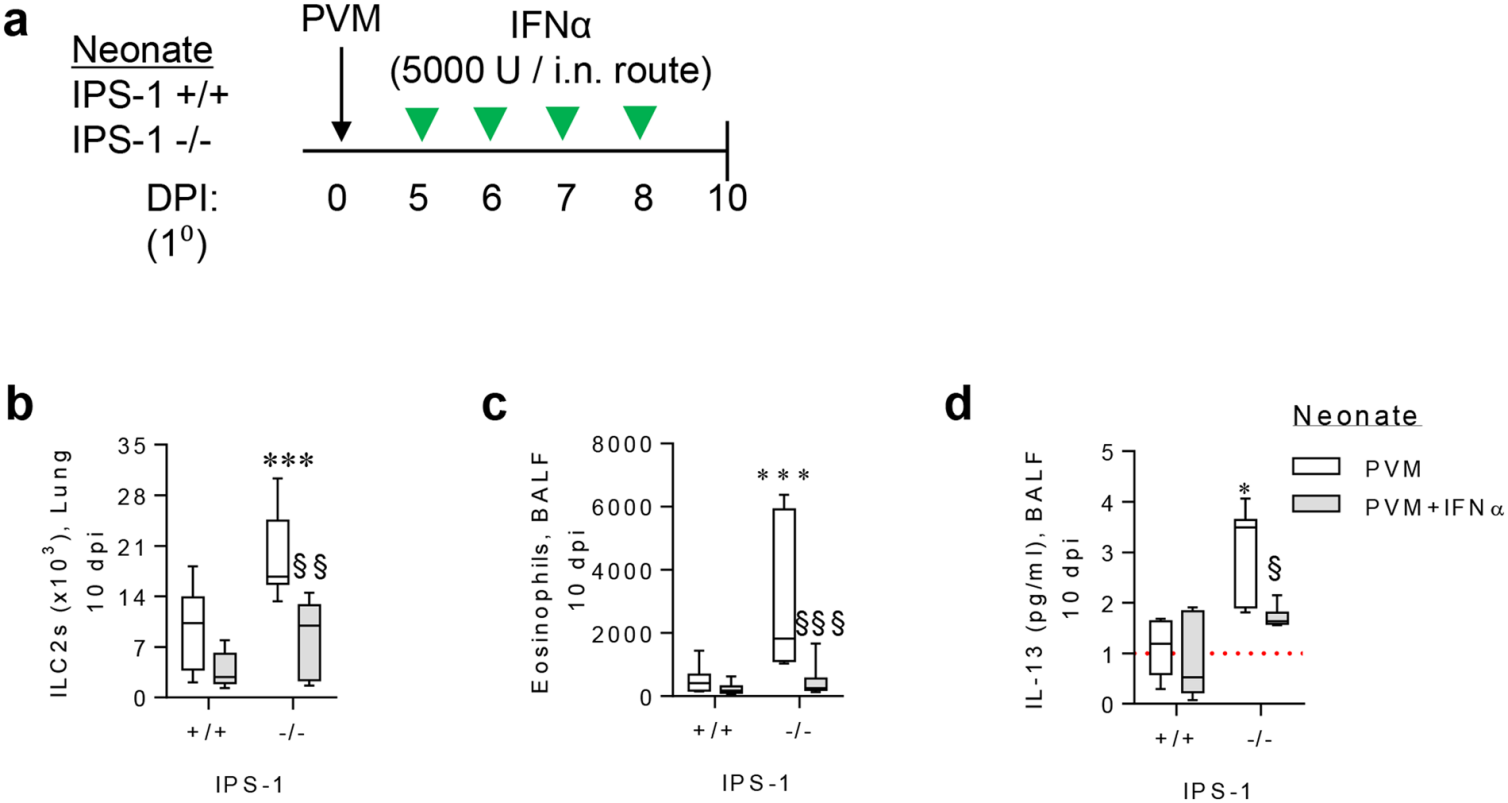
TLR7/IPS-1-induced IFN-α production negatively regulates type-2 inflammation in PVM infected IPS-1^−/−^ mice. (**a**) Study design for recombinant IFNα treatment of WT and IPS-1^−/−^ mice. (**b**) ILC2 number in lung (gated on Lin−, CD25+, ST2+, CD90.2+). (**c**) Eosinophils in BALF. (**d**) IL-13 protein expression in BALF. Experiments were performed twice with 8 to 12 mice per group. Data are mean and the standard error of the mean. *^,^**^,^***^,^****Denotes significance between WT and IPS-1^−/−^ infected mice. ^§,§,§§§^Denotes significance between PVM infected IPS-1^−/−^ mice treated with IFN-α and vehicle (PBS). Detection limits are denoted by a red dotted line.

Type I IFN is known to negatively regulate type-2 immunity. To test whether the absence of this counter-regulation contributed to the increased type-2 inflammation in bronchiolitic IPS-1^−/−^ mice, WT and IPS-1^−/−^ mice were treated daily from 5 to 8 dpi with recombinant IFN-α (i.n. route) and euthanized at 10 dpi (Fig. 8a). IFN-α administration significantly decreased the number of lung ILC2s (Fig. 8b), airway eosinophils (Fig. 8c) and IL-13 (Fig. 8d) production in IPS-1^−/−^ but not WT mice.

## Discussion

Severe RSV-bronchiolitis is a major cause of morbidity and an independent risk factor for the later development of childhood asthma^1, 5, 6^. In this study, we found that the absence of IPS-1 during primary viral infection increased viral burden and epithelial damage, leading to the release of tissue alarmins and the induction of type-2 inflammation and airway remodelling. Secondary infection of IPS-1^−/−^ mice but not WT mice recapitulated the type-2 inflammatory response and promoted the development of several cardinal features of a non-atopic asthma-like phenotype, highlighting a central role for IPS-1 in mediating resistance against both bronchiolitis and non-atopic asthma. Further, we identify that IPS-1-mediated signalling is critical for the recruitment of pDCs, leading to the generation of a IFN-α^high^/IL-33^low^HMGB1^low^ cytokine microenvironment in the lung that protects against bronchiolitis and subsequent asthma^17, 19^. Thus, the coordinated antiviral host response to pneumovirus infection requires both an RLR/IPS-1 signal to induce the recruitment of pDCs, as well as TLR7-mediated activation of the recruited pDC^18^, explaining the non-redundant role of both PRRs.

Impaired type-I IFN responses in both structural and immune cells, including pDCs, have been demonstrated in patients with asthma^33–35^, although this phenotype was not reproduced by some investigators, possibly reflecting genetic variation^36^. Polymorphisms at the *IPS1* locus are linked to type-I IFN deficiency in man and may therefore underlie susceptibility to viral infections^16^. Here, we questioned whether an interaction between IPS-1 deficiency and respiratory viral infection might underpin the association between attenuated IFN responses, severe bronchiolitis and subsequent development of asthma. We found that IPS-1 deficiency predisposed toward viral bronchiolitis and promoted the outgrowth of type-2 inflammation and airway remodelling. Central to this phenotype was a defect in antiviral cytokine production and its sequelae: a markedly greater viral load in the airway epithelium, which became necrotic, denuded and sloughed, and release of the alarmins IL-33 and HMGB1. This phenotype was accompanied by the expansion of ILC2s, type-2 cytokine expression (IL-5/IL-13), and ASM remodelling. Notably, this type-2 inflammatory phenotype did not occur in adult IPS-1 deficient mice infected with PVM suggesting that this response was age-dependent, mirroring the human epidemiology of susceptibility to RSV bronchiolitis.

The generation of type-2 immunity following the profound loss of epithelial barrier integrity is consistent with its teleological role in mediating wound repair and remodelling^37, 38^. ILC2s are now viewed as key regulators of tissue repair and homeostasis^39, 40^, and are the major ILC subfamily member present in the lungs, where they serve as tissue-resident sentinels awaiting alarm signal(s) indicative of barrier disruption. For example, the tissue damage in the lung that occurs during a helminth infestation leads to the release of IL-33, increasing the production of ILC2-derived type-2 cytokines, which in turn induce barrier repair and restore tissue homeostasis^41–43^. A similar process is thought to occur in the pathogenesis of asthma, except that the initiating insult is generally an aeroallergen or a respiratory virus, the latter causing considerable tissue damage, especially when the antiviral immune response of the host is impaired^17^. It is noteworthy that whereas type-2 cytokines, including IL-33, contribute to anti-helminthic immunity, they do not promote viral clearance; indeed they may compromise host defence by counter-regulating IFN responses in immune cells^19, 44^. This chain of events, starting in early-life and occurring repeatedly upon subsequent rounds of viral infections in susceptible individuals, may explain why asthma is frequently characterised by type-2 inflammation and remodelling of the airway wall. Consistent with this hypothesis, secondary infection of IPS-1^−/−^ mice led to a non-atopic asthma-like phenotype, including AHR, increased ASM mass, mucus hypersecretion, periostin deposition and eosinophilic inflammation. Using this same infection model, we found previously that TLR7^−/−^ mice are similarly predisposed to virus-induced asthma-like pathologies^17^. Interestingly, TLR7^−/−^ mice that had developed severe viral bronchiolitis in early-life were also at greater risk of sensitization to a bystander allergen. Whether severe bronchiolitis in IPS-1^−/−^mice predisposes to allergic sensitization remains to be tested.

IPS-1 deficiency increased viral burden in the epithelium and delayed viral clearance. This was associated with increased TNF-α production in the lungs, sloughing of the airway epithelium, and alarmin release. TNF-α peaked early in the lungs of IPS-1^−/−^ mice in response to infection and was associated with the infiltration of neutrophils in the airway lumen prior to the onset of AEC sloughing. Paradoxically, in addition to inducing survival, TNF-α receptor signalling can induce different modes of cell death, including apoptosis and necroptosis, and this can profoundly influence whether the ensuing response is pro- or anti-inflammatory^45, 46^. *Caspase-3*, *Rip1*, *Rip3* and *Mlkl* gene expression and cleaved caspase-3 and MLKL protein expression in the airway epithelium were all significantly elevated in the lungs of virus-infected IPS-1^−/−^ neonates, indicating that both apoptosis and necroptosis were occurring in response to the same stimulus, as recently described in influenza infection^47^. The increase in *Rip1* expression was not as prominent as *Rip3*, however necroptosis can proceed in a RIPK1-independent, RIP3 and MLKL-dependent manner^46, 48^. The trigger for necroptosis in IPS-1^−/−^ mice remains unknown, but possibilities include TNFR, TLR4 or TLR3 signalling^48–53^, the latter induced by an elevated viral load, which was evident in the IPS-1^−/−^ mice. It is noteworthy that necroptosis has been linked to the release of nuclear alarmins^54–56^, as peak expression of *Rip3* and *Mlkl* occurred at the same time as peak HMGB1 and IL-33 release in IPS-1^−/−^ mice. These alarmins were associated with ILC2 expansion and type-2 cytokine production, which in turn, can induce features of repair and airway remodelling. Thus, by facilitating the release of alarmins, necroptosis may act to promote type-2 immunity and tissue repair following excessive tissue damage. Future studies exploring the effect of necroptosis inhibitors such as necrostatin-1s are warranted to investigate this concept.

We have previously shown that TLR7-mediated recognition of PVM by pDCs plays a critical, non-redundant role in initiating host defence^17, 18^. This was somewhat unexpected given the ability of other PRRs to recognise viral-associated molecular patterns and elicit type-I IFN production^57^. Indeed, other investigators have demonstrated that IPS-1, not utilised in the TLR7 signalling pathway, contributes to the production of type-I IFN in mice following inoculation with hRSV^20–22^. Here, we found that in response to PVM infection, IPS-1 deficiency impaired the production of IFN-α in the airway, a finding that is broadly consistent with the observations from hRSV inoculated IPS-1^−/−^ mice^20–22^. Because PVM is a mouse pathogen, one can employ a low inoculum and follow host-pathogen interactions over time. Using this strategy, we have reproducibly shown that type-I IFNs peak at 7 dpi, and that this response is dependent on TLR7-induced activation of pDCs^17–19^ Consistent with a central role for pDC in the generation of type-I IFN in response to pneumoviruses^58, 59^ we found that the attenuated IFN-α response in IPS-1^−/−^ mice was associated with a greater than 70% reduction in pDCs in the lung prior to and at the peak time of PVM infection. Interestingly, administration of a TLR7 agonist to IPS-1^−/−^ mice failed to replicate the induction of *Irf7*, *Viperin* or *Stat1* mRNA expression observed in TLR7 sufficient WT mice, suggesting that TLR7 and IPS-1 signalling cooperate in a linear manner to promote antiviral immunity against PVM. Although the levels of IFN-λ protein were unaffected by the absence of IPS-1, not one of the IFN-stimulated genes that we measured was up-regulated in response to PVM infection in IPS-1^−/−^ mice, inferring that these genes were primarily regulated by IFN-α. The impaired pDC/IFN-α response may also have contributed to the elevated type-2 inflammation^19, 60^. In support of this idea, we found that supplementation of IPS-1^−/−^ mice with exogenous IFNα reduced ILC2 numbers, airway eosinophilia and IL-13 production. Although we did not explore the molecular link between IPS-1 signalling and pDC recruitment, several chemokines are up-regulated downstream of IPS-1/NFκB signalling^61–65^, and many of these are able to recruit pDCs^66^. Additionally, the chemoattractant chemerin also contributes to pDC recruitment. Indeed it is noteworthy that mice deficient in the chemerin receptor Chem R23 have fewer lung pDCs, impaired IFN-α and IL-12 production, higher viral load and present with a severe neutrophilia during acute PVM infection^67^. Since chemerin is expressed at high concentration at baseline and regulated by proteolytic activation, it is possible that IPS-1 signalling contributes to pDC recruitment by regulating the expression of a protease. Nevertheless our findings that IPS-1 signalling is required to increase pDCs in the lung provides a possible mechanism to explain the non-redundant role of both the RLR/IPS-1 signalling and TLR7/MyD88 signalling in the generation of optimal host defence against pneumovirus infection.

In summary, we demonstrate a possible gene-environment interaction that increases host susceptibility to severe virus-associated bronchiolitis and the onset of non-atopic asthma. IPS-1 signalling contributes to the recruitment of pDCs which are critical for effective antiviral immunity and the prevention of immunopathology. The IL-33 and HMGB1-rich cytokine microenvironment that occurs as a consequence of airway epithelial cell death skews the ensuing inflammatory response toward a type-2 response, leading to increased ASM growth in early-life and potentially long-term changes in both resident airway cells and immune cells necessary for progression to a non-atopic asthma-like phenotype following viral challenge in later-life.

## Materials and Methods

### Mice, treatments and PVM infection

Specific pathogen-free wild-type (WT) and IPS-1-deficient (IPS-1^−/−^) C57BL/6 mice^68^ were housed in individually ventilated cages and intranasally inoculated at 7 days of age with 2 plaque-forming units (PFU) of PVM (J3666 strain)^17, 18^ in 10 µl of vehicle (10% foetal calf serum in DMEM media) or vehicle. In some experiments, mice were re-inoculated with 100 PFU of PVM in 50 μL of vehicle or vehicle 42 dpi. To examine TLR7 signalling and the effect of exogenous type-I IFN, neonatal mice were intranasally administered 15 µg of imiquimod or 5000 IU of recombinant IFN-α in 10 uL of PBS or vehicle diluent as per the study designs in Figs 7f and 8a, respectively. This study was approved by the University of Queensland Animal Care and Ethics Committee and performed in accordance with the relevant guidelines and regulations.

### Bronchoalveolar lavage fluid (BAL)

BAL was performed and cells prepared as previously described^17, 19^. Briefly, PBS (400 µL for neonates; 600 µL for adults) was washed through the lungs using the tubing and adapter from a 20 G catheter (BD) inserted into the trachea in adult mice. For BAL of neonatal mice, a catheter adapter outfitted with 0.1 mm diameter vinyl tubing was used. After collection, the bronchoalveolar lavage fluid (BALF) centrifuged at 5000 rpm. The supernatant was stored at −20 °C and used to determine cytokine production. BAL cells were resuspended in Gey’s red blood cell lysis buffer (home-made) for 5 min on ice to remove erythrocytes. The remaining leukocytes were counted using a haemocytometer and stained with an antibody cocktail as described below.

### Flow cytometry

Lung cells were mashed through a cell strainer and red blood cells lysed with Gey’s red blood cell lysis buffer. Lung or BAL leukocytes were seeded into a U bottom 96 well plate at 10^6^/well, and pre-incubated with anti-FcγRIII/II (‘Fc-block’) in PBS/1% FCS medium prior to a 30 minute incubation with one or more of the following fluorochrome-labelled antibodies (BD Biosciences unless otherwise stated). Antibodies used include: CD11c-FITC, SiglecH-AF647, B220-V500, CD11b-PerCP Cy5.5. Ly6G-AF488 Siglec-F-APC, B220-PE, CD11b-PerCP Cy5.5, CD3-PE, MHCII-APC-Cy7 CD11c-FITC. CD45R-AF488, CD3-AF488, CD11c-AF488, CD2-AF488, Gr-1-AF488, CD11b-AF488, CD90.2-APC-Cy7 and CD25-BV650. After three washes in PBS/1% FCS medium, stained cells were analysed using a BD LSR Fortessa X-20.

### Measurement of airway hyper-reactivity (AHR)

AHR was measured 7 days after re-infection with PVM by forced oscillation technique using Flexivent apparatus (SCIREQ, Montreal, Canada) as described previously^69^. Briefly, mice were anesthetized by injecting a cocktail of xylazine (0.2 mg/10 gm of body weight) and ketamine (0.4 mg/10 gm of body weight) and maintained under mechanical ventilation. Airway resistance was measured at increasing doses of nebulised methacholine (0 to 30 mg/ml for 1 min) (Sigma-Aldrich, St Louis, MO, USA).

### ELISA, cytokine bead array (CBA) and dsDNA assay

The concentration of TNF-α, IL-33, IFN-λ, IFN-γ, IL-5, IL-6, IL-12p40, IL-17A (R&D systems, Minneapolis, MN, US) and HMGB1 (Chondrex, Redmond, WA, US) expression in BALF and/or lung homogenates was analysed by ELISA according to the manufacturer’s protocol. Cytokine bead array (CBA) was used to measure IL-13 (BD) and ProcartaPlex Mouse IFN alpha/IFN beta was used to measure IFN-α/β (ebioscience) as per the manufacturer’s instructions. dsDNA in BALF was analysed by Quant-iT PicoGreen dsDNA Assay Kit (ThermoFisher) according the manufacturer’s protocol

### Quantitative real time PCR

Total RNA was isolated from the inferior right lung lobe with TriReagent solution (Ambion) followed by phenol-chloroform extraction. DNAse digestion was performed with Turbo DNAse (Ambion), according to the manufacturer’s instructions. Reverse transcription was performed using M-MLV reverse transcriptase and random primers (Invitrogen). qRT-PCR was performed with SYBR Green (Life Technologies) with primers listed in Supplementary Materials, Table S1. Expression values were normalized relative to the housekeeping gene *Hprt* and expressed as fold change relative to vehicle control mice of the same genotype using the 2^−ΔΔCT^ formula.

### Immunohistochemistry and histology

Paraffin-embedded sections were prepared as previously described^70^. Immunohistochemistry for PVM, HMGB1, α-smooth muscle actin, TGFβ, MLKL, cleaved caspase-3 and periostin occurred as described previously^19, 70^. Briefly, slides were dehydrated using xylene and ethanol washes before antigen retrieval using citrate buffer. Lung sections were permeabilized using 0.5% Triton in PBS for 10 minutes. Non-specific binding was blocked with 10% normal goat serum in PBS. Primary antibodies used include anti-PVM (kindly provided by Dr. Ulla Buchholz, Laboratory of Infectious Diseases, National Institute of Allergy and Infectious Diseases), anti-HMGB1 (Abcam; 1:400 dilution), anti-α-smooth muscle actin (Sigma; 1:800), anti-TGF-β (Abcam; 1:200), anti-MLKL (Merk Millipore; 1:200), cleaved caspase-3 conjugated-488 (CST; 1:200) and anti-perisotin (Abcam; 1:200). Tissue sections and primary antibodies were incubated overnight at room temperature. With the exception of MLKL and caspase-3, sections were washed 3 times in PBS/Tween 20 before 60 minutes of incubation with either anti-rabbit IgG-AP (Sigma; 1:200), anti-mouse IgG-AP (Sigma; 1:200) or anti-rat IgG-AP (Sigma; 1:200). After washing, color was developed with Fast Red substrate (Sigma). Sections were counterstained with hematoxylin before mounting with Glycergel (Dako). For immunofluorescence (MLKL and cleaved caspase-3-488), MLKL-stained sections were incubated with goat anti-rat-647 for 60 mins. After washing with 0.5% Triton in PBS, sections were counterstained using DAPI (Sigma; 1:10,000), mounted with fluorescent mounting media (DAKO) and imaged on Diskovery Spinning Disk confocal microscope (Nikon).

### Statistical analysis

Data presented are the mean ± s.e.m. Data sets were analysed by Student *t* test for parametric data and Mann-U Whitney test for non-parametric data except for time course experiments which were analysed by two-way ANOVA and Bonferroni Post Hoc Test. GraphPad Prism 6.01 (GraphPad Software) was used for all data analysis and preparation of graphs.

## Electronic supplementary material

Supplementary Information
