# Supplementary material for: The Absence of Interferon-β Promotor Stimulator-1 (IPS-1) Predisposes to Bronchiolitis and Asthma-like Pathology in Response to Pneumoviral Infection in Mice
Source: Sci Rep. 2017 May 24;7:2353. doi: 10.1038/s41598-017-02564-9 (PMC5443759; doi:10.1038/s41598-017-02564-9)
Supplement: Supplementary file 1 — Supplementary Information [file 41598_2017_2564_MOESM1_ESM.pdf]

Supplementary Information

The Absence of Interferon-β Promotor Stimulator-1 (IPS-1) Predisposes to Bronchiolitis and an Asthma-like Pathology in Response to Pneumoviral Infection in Mice

Jennifer Simpson[1]*, Jason P. Lynch[1]*, Zhixuan Loh[2]*, Vivian Zhang[2], Rhiannon B. Werder[1], Kirsten Spann[3,4,5] , Simon Phipps[1,5].

* Authors contributed equally

[1]School of Biomedical Sciences, The University of Queensland, Queensland 4072, Australia;

[2]Institute of Molecular Biosciences, The University of Queensland, Queensland 4072, Australia;

[3] School of Biomedical Sciences, Queensland University of Technology 4001, Australia;

[4]Child Health Research Centre, Institute of Health and Biomedical Innovation 4006, Australia;

[5]Australian Infectious Diseases Research Centre, The University of Queensland, Queensland, Australia;

[1] **Correspondence and reprints**

Dr. Simon Phipps, School of Biomedical Sciences, University of Queensland, QLD, Australia, St. Lucia, QLD, Australia.

s.phipps@uq.edu.au phone 61(0)733652785 fax 61(0)733651766.

[2] **Funding**

This work was supported by National Health and Medical Research Council of Australia Project grant to S.P., and an Australian Research Council Future Fellowship to S.P.

Supplementary Table 1: Oligonucleotide primer sequences are shown.

Supplementary Figure 1: Representative flow cytometry plots showing the gating of ILC2 in lung. Quadrants display % of total population.

Supplementary Figure 2: Absence of IPS-1 in adult mice does not predispose to airway eosinophilia or HMGB1 release in response to PVM infection. 6-week-old WT or IPS-1$^{-/-}$ mice were infected with 2 pfu in early life. (A) Eosinophil numbers in BALF. (B) Quantification of cytoplasmic HMGB1.

Supplementary Figure 3: Absence of IPS-1 does not predispose to elevated type-1 or type-17 inflammation. (A) IFN-γ protein expression in BALF. (B) IL-17A protein expression in BALF. (C) Neutrophil number in BALF. (D) Mononuclear cell number in BALF. (E) Total BALF cells. Data are mean and the standard error of the mean. *, **, ***,**** denotes significance between WT and IPS-1$^{-/-}$ infected mice. #, ##, ### denotes significance between infected and naïve IPS-1$^{-/-}$ mice. †, ††, ††† denotes significance between naïve and infected WT mice.

Sup Table 1

**Gene**	**Forward Primer (5’-3’)**	**Reverse Primer (5’-3’)**
Hprt	AGGCCAGACTTTGTTGGATTTGAA	CAACTTGCGCTCATCTTAGGCTTT
Rip1	CTCCAACACACCACTTTTGG	CTCCAACACACCACTTTTGG
Rip3	TCCCAATCTGCACTTCAGAAC	GACACGGCACTCCTTGGTAT
Mlkl	AGGAACCAGTGGGTCAGGAT	CAAGATTCCGTCCACAGAGGG
Caspase 3	TGAATCCACTGAGGTTTTGTTG	TGCTGGTGGGATCAAAGC
Irf7	CTTAGCCGGGAGCTTGGATCTACT	CCCTTGTACATGATGGTCACATCC
Viperin	AACCTGCTCATCGAAGCTGT	ATAGTGAGCAATGGCAGCCT
Stat1	TTTGATGAGATGTCCCGGATAGT	TCGCCAGAGAAAATTCGTGTTT
PVM *SH*	GCCTGCATCAACACAGTGTGT	GCCTGATGTGGCAGTGCTT

Sup fig 1

a

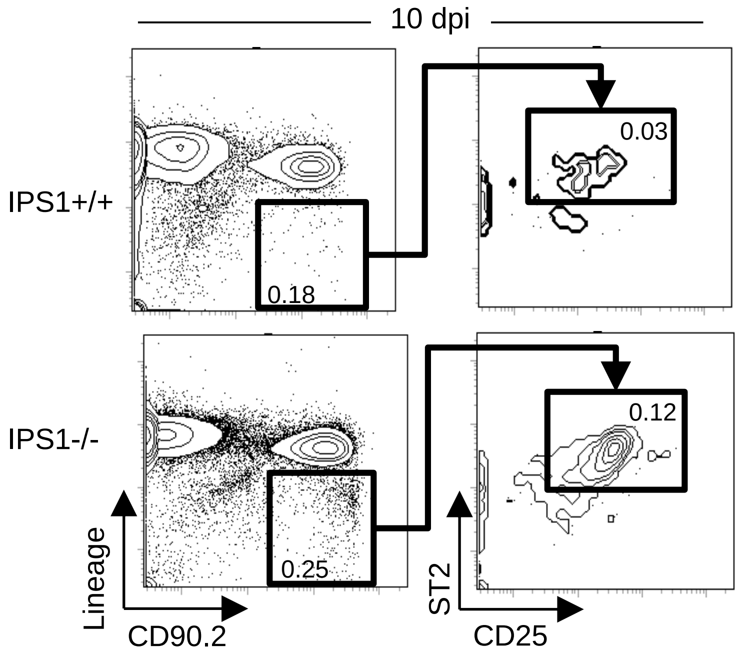

Sup fig 2

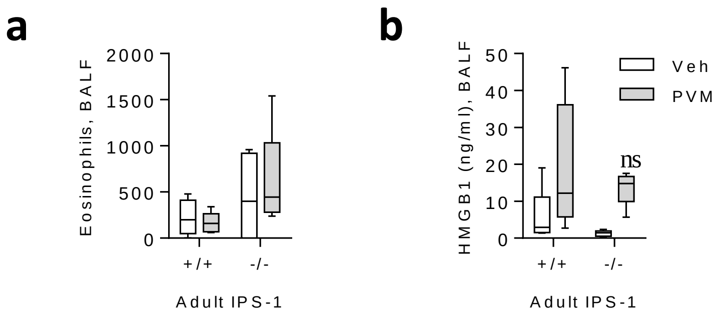
a
Eosinophils, BALF
2000
1500
1000
500
0
+/+
-/-
Adult IPS-1
b
HMGB1 (ng/ml), BALF
50
40
30
20
10
0
Veh
PVM
ns
+/+
-/-
Adult IPS-1

Sup fig 3

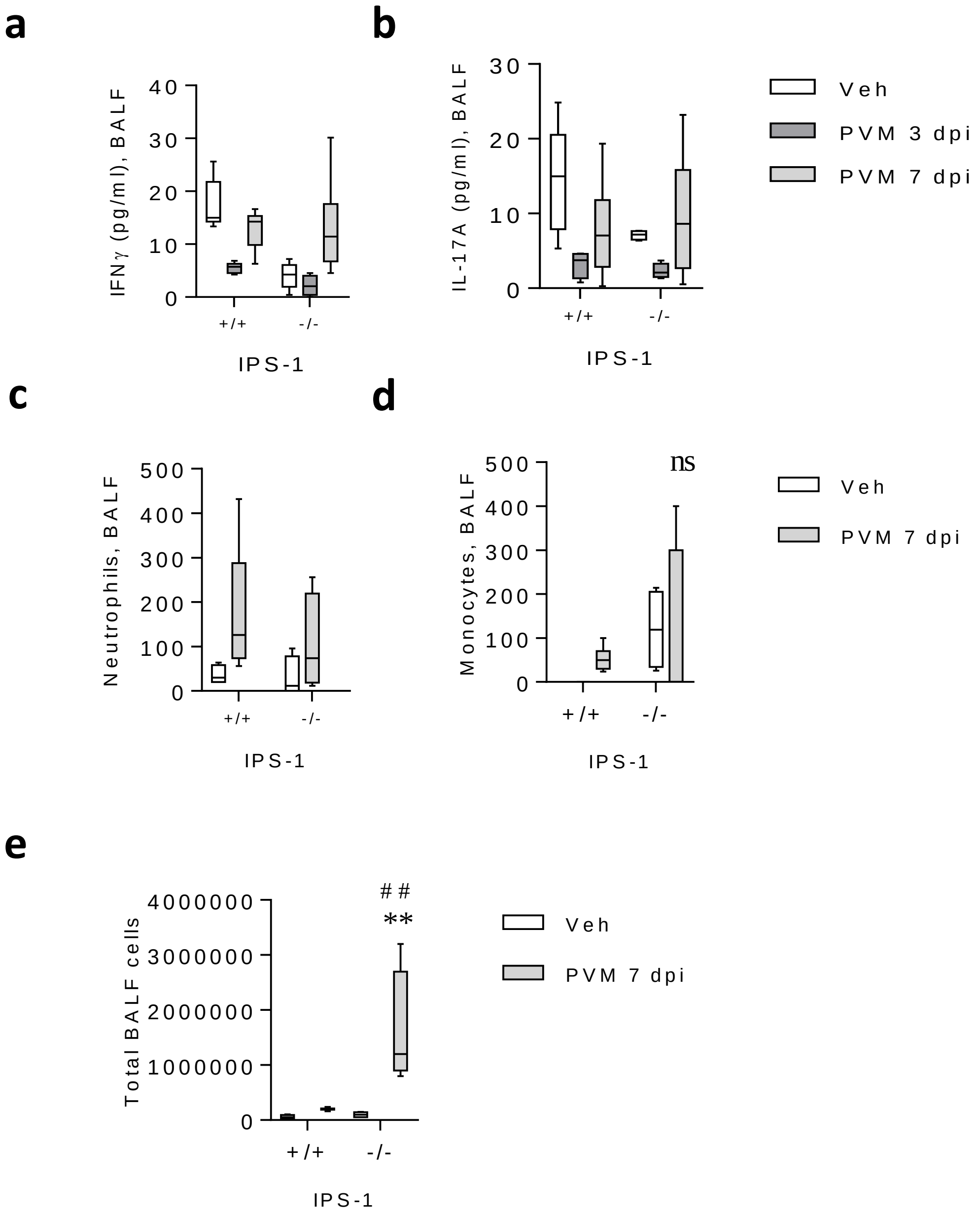
a
b
c
d
e
IFNγ (pg/ml), BALF
0
10
20
30
40
+/+
-/-
IPS-1
IL-17A (pg/ml), BALF
0
10
20
30
Veh
PVM 3 dpi
PVM 7 dpi
Neutrophils, BALF
0
100
200
300
400
500
Monocytes, BALF
ns
Veh
PVM 7 dpi
Total BALF cells
0
1000000
2000000
3000000
4000000
##
**
Veh
PVM 7 dpi
